# Regarding: “Associations between the completion rate of the unsupervised community swallowing training and swallowing function in older adults with mild dysphagia: a quasi-experimental pre-post study without control’

**DOI:** 10.1080/07853890.2026.2626068

**Published:** 2026-02-02

**Authors:** Youtao Zhang

**Affiliations:** The First Affiliated Hospital of Hebei North University, Zhangjiakou, Hebei Province, 075000, China

To the Editor,

The investigation by Wang et al. into the unsupervised Simple Swallowing Training Program (SSTP) addresses a vital epidemiological imperative: scalable, community-based management of presbyphagia [1]. While establishing a training threshold of ≥20 sessions offers a practical clinical heuristic, the translational potential of these findings is constrained by traditional analytical choices. To elevate such studies from observational feasibility trials to precision medicine benchmarks, we propose three specific methodological advancements tailored to the complexities of unsupervised geriatric rehabilitation.

First, the determination of the minimum effective frequency relied on discretizing continuous training volume into 31 subgroups based on session counts. While intuitively simple, binning continuous variables dilutes statistical power and increases the risk of multiple comparison artifacts, necessitating heavy corrections like the Bonferroni method. Future analyses should prioritize Segmented Regression or Change Point Analysis [2]. These methods mathematically identify the physiological ‘structural break’ – the exact dosage where recovery slopes shift – rather than relying on the statistical significance of fragmented bins. This approach is increasingly standard in determining precise dosage thresholds in rehabilitation sciences.

Second, adherence was defined strictly by binary self-reporting (‘Yes/No’) without verification. In geriatric rehabilitation, ‘completion’ does not equate to ‘correct execution’. Unsupervised older adults frequently adopt compensatory movement patterns that may mask true functional gains or induce fatigue. To mitigate this fidelity gap, future protocols must incorporate digital biomarker validation or Computer Vision [3]. Recent advancements in remote monitoring demonstrate that analyzing even a random subset of sessions *via* smartphone cameras can verify movement amplitude and technique, ensuring that reported adherence reflects high-quality physiological engagement rather than mere task completion.

Finally, the cohort presented mild dysphagia with a median baseline Modified Barium Swallow Impairment Profile (MBSImP) score of 6.0. While MBSImP is the gold standard for diagnosing impairment, it may lack the sensitivity to detect micro-level physiological adaptations in populations near the measurement ‘floor’. This measurement ceiling likely explains the non-significant esophageal findings. Integrating continuous quantitative bio-metrics – such as lingual pressure measurement or surface electromyography (sEMG) – would capture the subtle neuromuscular adaptations that precede visible radiological changes [4].

While we fully acknowledge the practical importance of Wang et al’.s observation that higher training completion correlates with better outcomes, it is critical to consider how methodological choices may influence the interpretation of effect size and clinical relevance. The binary definition of adherence and the use of coarse-grained subgroup analyses may lead to a modest overestimation of the threshold effect, while the insensitivity of MBSImP to micro-level adaptations may obscure early functional gains. Future studies incorporating continuous, objective metrics may therefore refine – not necessarily negate – the promising trends reported here.

Implementing these precision analytics and fidelity mechanisms will not only help validate the SSTP but also establish a rigorous benchmark for interpreting and replicating such findings in the next generation of community-based rehabilitation research, ultimately enhancing care for elderly patients with dysphagia.

## Data Availability

There is no data associated with this research.
